# The Prince and Princess See the Stamps Printed

**Published:** 1897-06-05

**Authors:** 


### THE PRINCE AND PRINCESS SEE THE STAMPS PRINTED.

The Prince and Princess of Wales, accompanied by Princess Victoria, paid a visit on Friday afternoon, 28th ult., to the works of Messrs. De La Rue and Co. in order to see the process of printing the hospital stamps. Lady Emily Kingscote and Captain Holford were in attendance. The Royal party were received at the entrance in Dufferin Street by Mr. T. Andros De La Rue (the chairman of the company), Mr. Ernest De La Rue, and other directors, and proceeded to that portion of the works which has been set apart solely for the production of these stamps. On arriving there, the process employed was explained by Mr. T. Andros De La Rue, and the printing of several sheets of stamps was closely watched by the Royal visitors, one of the sheets thus printed being initialled by His Royal Highness as a memento of his visit. The Prince and Princess of Wales and Princess Victoria signed the visitors' book, and the Prince before leaving addressed those present as follows: "Before going I wish to say how very much interested we have been in seeing this process of printing the stamps. I most earnestly hope that the working classes will buy as many of them as possible, because by doing so they will be afforded an opportunity of giving a shilling to the Hospital Fund, and they will always have a souvenir of the occasion." Amongst those present were Lord Rowton, Sir Savile Crossley, Bart., Mr. Henry C. Burdett, Mr. Purcell, Mr. Stuart Wortley, M.P., and Mr. J. G. Craggs, the two latter of whom were presented by the Prince to the Princess.

